# Adsorption of phosphate on iron oxide doped halloysite nanotubes

**DOI:** 10.1038/s41598-019-39035-2

**Published:** 2019-03-01

**Authors:** Dema A. Almasri, Navid B. Saleh, Muataz A. Atieh, Gordon McKay, Said Ahzi

**Affiliations:** 1 Qatar Environment and Energy Research Institute (QEERI), Hamad Bin Khalifa University (HBKU), Qatar Foundation, PO Box 34110, Doha, Qatar; 2 College of Science and Engineering, Hamad Bin Khalifa University, Qatar Foundation, PO Box 34110, Doha, Qatar; 3 Department of Civil, Architectural and Environmental Engineering, University of Texas, Austin, TX 78712 USA

## Abstract

Excess phosphate in water is known to cause eutrophication, and its removal is imperative. Nanoclay minerals are widely used in environmental remediation due to their low-cost, adequate availability, environmental compatibility, and adsorption efficiency. However, the removal of anions with nanoclays is not very effective because of electrostatic repulsion from clay surfaces with a net negative charge. Among clay minerals, halloysite nanotubes (HNTs) possess a negatively charged exterior and a positively charged inner lumen. This provides an increased affinity for anion removal. In this study, HNTs are modified with nano-scale iron oxide (Fe_2_O_3_) to enhance the adsorption capacity of the nanosorbent. This modification allowed for effective distribution of these oxide surfaces, which are known to sorb phosphate via ligand exchange and by forming inner-sphere complexes. A detailed characterization of the raw and (Fe_2_O_3_) modified HNTs (Fe-HNT) is conducted. Influences of Fe_2_O_3_ loading, adsorbent dosage, contact time, pH, initial phosphate concentration, and coexisting ions on the phosphate adsorption capacity are studied. Results demonstrate that adsorption on Fe-HNT is pH-dependent with fast initial adsorption kinetics. The underlying mechanism is identified as a combination of electrostatic attraction, ligand exchange, and Lewis acid-base interactions. The nanomaterial provides promising results for its application in water/wastewater treatment.

## Introduction

Phosphorus is an essential micronutrient for many aquatic organisms; however, its availability in excess can compromise the health of the natural environment^1^. Specifically, excess phosphorus can cause harmful algal blooms, hypoxia in water, stresses on aquatic life, and can also compromise drinking water quality (e.g., odor and taste)^2^. To regulate eutrophication, the United States Environmental Protection Agency (USEPA) recommends 0.05 and 0.1 mg/L of total phosphate in streams entering lakes and in flowing water bodies, respectively^3^. Removal of excess phosphate from water is thus imperative.

To-date, various biological, chemical, and physical treatment processes have been developed for the removal of dissolved phosphate^4^. Biological processes, such as conventional activated sludge, can remove >97% of phosphate from water; however, these processes are less efficient in removing trace amounts of phosphate^5^. Precipitation (e.g., as struvite), a simple but effective chemical treatment process, is limited by the complexity in sludge handling, neutralization, waste disposal, and treatment and management costs^5,6^. Physical separation techniques, such as reverse osmosis or electrodialysis, have been shown to be expensive and ineffective (remove only 10% of the total phosphate)^7^. Also, conventional ion exchange processes can remove anions; however, the preference of the anion exchange resins for sulfates (over phosphates), which are present in higher concentrations in wastewater (compared to phosphate), compromises their efficiency for phosphate removal^8^. Adsorption is reported to be one of the most effective processes for phosphate removal with advantages of low-cost, high efficiency (a wide range of concentration), and simple operation^9^.

A variety of adsorbents with unique sorption properties and surface regeneration-abilities have been studied. Activated carbon is commonly used due to its high efficiency and large surface area; however, it has a higher cost relative to other adsorbents^10^. Recently, the development of low-cost adsorbents such as iron hydroxides^11^, alum sludge^12^, and sand^13^ have been gaining interest for aqueous phosphate removal. While these low cost-alternatives are available, many suffer from low phosphate removal efficiency, sorbent-particle agglomeration, or difficulty in sorbent separation from water^13–15^. A major focus has been on clays due to their relative abundance, environmental compatibility, and adsorption efficiency^16^. The natural abundance of clay and its low-cost allow for bypassing the sorbent regeneration aspect when these are spent; which is a unique advantage over the other more commonly used materials, e.g., activated carbon^17^.

Halloysite nanotube (HNT) is a type of naturally occurring aluminosilicate with a nano-tubular structure that has a negatively charged exterior and a positively charged internal lumen. The HNT chemical structure is similar to kaolin (i.e., Al_2_Si_2_O_5_(OH)_4_·*n*H_2_O), and is comprised of octahedral alumina crystals in the inner layer and a tetrahedral silica in the outer layer^16^. Its attractive features include high specific surface area, availability of micropores, presence of positive and negatively charged surface sites, and low-cost^18^. These attributes allow for HNTs to be integrated into various applications; e.g., HNTs are used as reinforcement fillers in polymers^19^, as drug delivery agents^20,21^, and as nanoreactors/nanotemplates for the synthesis of functional materials^22^ as well as for applications in environmental remediation^23,24^. The use of HNT as a viable sorbent has recently gained interest, and these tubular minerals have been shown to remove various heavy metals from water^23^.

There is a paucity of studies on removal of anionic phosphate species with HNT. The unique surface properties (i.e., the positively charged inner lumen) of HNTs render it to be a promising material for electrostatic removal of phosphates and other anions. The exterior of HNT is considered to be chemically inert, except for the hydroxyl functional groups on the sides and on the broken edges. These exterior negative functional moieties can present electrostatic repulsion to anions, and thus can decrease the efficacy of phosphate sorption. Shielding such negatively charged sites with a highly absorptive oxide surface, such as hematite, can be advantageous.

Hematite, a major component of fine-grained sediments, is known to have a strong affinity for phosphate anions. Chemical modification with hematite can increase the number of active sites on HNT exteriors, improve the ligand exchange properties, and may present new functional groups that favor phosphate adsorption^25,26^. Specifically, the ligand exchange process allows for adsorption of phosphate onto hematite surfaces. Relatively strong inner-sphere complexes form with hematite surface sites and phosphate, and allow for specific interactions between the sorbate and the sorbent sites^27–30^. The study design thus considers distributing hematite surfaces by an *in-situ* growth of these oxides onto nano-scale HNT exterior. This way, aggregation of hematite (with no other surface modification) can be avoided, which can lead to an improvement in adsorption capacity (for phosphate) of these tubular minerals^25,31,32^. Hematite, alongside with clay, is also a low-cost and environmentally benign material and thus is an attractive choice for a sorbent^33^. A synergistic removal of phosphate by a positively charged HNT interior and iron oxide modified exterior could prove to be a promising low-cost nano-sorbent alternative.

This study presents a facile synthesis of Fe_2_O_3_-HNTs with benign precursors and in mild reaction conditions. No previous study to-date has reported surface modification of HNTs with ferric oxides. The nanomaterials are characterized with scanning electron and scanning transmission electron microscopy (SEM and STEM), transmission electron microscopy (TEM), BET surface area analysis, X-ray fluorescence (XRF), X-ray diffraction (XRD), and surface charge analysis. The application of raw and hybridized HNTs for phosphate removal from water is reported. The effects of sorbent dosage, contact time, pH, initial phosphate concentration, and coexisting ions have been systematically studied. Well-known sorption kinetics and equilibrium sorption models are used to assess the underlying mechanisms of adsorption.

## Experimental Section

### Materials

All solutions were prepared with reagent grade chemicals and deionized water (Milli-Q system). Iron (III) chloride hexahydrate (FeCl_3_·6H_2_O) and ammonia were obtained from SureChem (Suffolk, U.K.) and VWR Chemicals (Leuven, Belgium), respectively. Glacial acetic acid was purchased from Fisher Scientific (Fair Lawn, NJ, U.S.A). HNTs and monopotassium phosphate (KH_2_PO_4_) were procured from Sigma-Aldrich Company Ltd. (Saint Louis, MO, U.S.A.).

### Preparation of modified HNT (Fe-HNT)

A modified sol-gel method was used to surface modify HNTs. In brief, 3 g of HNT was dispersed in 300 mL Milli-Q water and was magnetically stirred for 1 h. A desired amount (corresponding to the percent iron loadings) of FeCl_3_·6H_2_O was dissolved in 150 mL Milli-Q water. A diluted ammonium solution was added to the iron solutions so that the moles of hydroxides (from NH_4_OH) are three times to the moles of iron in the solution and could achieve appropriate ratio to form hydroxyiron (Fe(OH)_3_). The hydroxyiron solution was then added drop-wise to the HNT mixture and mixed at 350 rpm for 24 h. The mixture was then separated by centrifugation and washed with Milli-Q water several times prior to drying in air, overnight. The dried sample was collected and exposed to glacial acetic acid vapors in a furnace at 80 °C for 2 h. After exposure, the sample was left to dry at the same temperature for 30 min to remove any surface-sorbed acetic acid. The sample was then calcinated at 400 °C for 1 h to obtain the Fe-HNT. Finally, the cooled sample was sieved (100 μm sieve) to remove any sintered or agglomerated fraction. The Fe-HNT samples, prepared at different iron loadings of 0.25, 0.5, 1, and 5 wt.%, are designated in the text as 0.25Fe-HNT, 0.5Fe-HNT, 1Fe-HNT, and 5Fe-HNT, respectively.

### Characterization

The surface morphology of the samples was studied with a JEOL JSM-7610F field emission SEM at an accelerating voltage of 5 kV. TEM images were obtained by placing the sample on lacey carbon film using an FEI Talos F200X TEM, and operating the TEM at 200 kV; the TEM is equipped with an STEM and an energy dispersive X-ray spectroscope (EDX). The specific surface area of HNT and modified HNT was measured with a Micromeritics ASAP 2020 BET N2 (Norcross, GA, U.S.A.) surface area analyzer at 77 K. A Rigaku ZSX Primus II Wavelength Dispersive XRF (Austin, TX, U.S.A.) was used to perform elemental analysis, while crystallinity was analyzed with a Rigaku Miniflex-600 XRD (Chapel Hill, NC-U.S.A.), equipped with Cu-Kα lamp (λ = 0.154 nm). Surface charge was measured with a Mobius (Santa Barbara, CA, U.S.A.) zeta potential analyzer.

### Aqueous Phosphate Analysis

Aqueous samples were analyzed for phosphate with an ion chromatography (IC) unit (Dionex ICS5000). Standards with known phosphate concentration were analyzed to obtain a calibration curve to ensure accuracy in analysis. At the end of the adsorption experiments, the samples were filtered using a 0.22 µm filter and a small aliquot was diluted with DI water at a desired concentration range (between 1–100 ppm) for analysis with IC.

### Batch Adsorption Protocol

Unless stated otherwise, 3 g L^−1^ of the adsorbent was placed in a centrifuge tube with a 10 mg L^−1^ phosphate solution. The pH of the solution was adjusted with 0.1–1 mg L^−1^ HCl or NaOH. All samples were mechanically mixed on a shaker table (at 350 rpm in HBKU, Qatar and at 240 rpm in Austin, TX) at room temperature. For the kinetics and adsorbent dosage studies, the pH of the initial phosphate solution (i.e., pH of 5.0) was not altered (to avoid external chemical perturbation). The most efficient sorbent dose was determined from studies performed with sorbent amount ranging between 0.1 to 8.0 g L^−1^. The sorbent dose used for the kinetics and equilibrium experiments was 3 g L^−1^ as this was found to be the most efficient amount for phosphate removal (Fig. S1). Kinetic experiments were conducted at time intervals ranging between 0.5 to 240 min to determine the equilibrium contact time and maximum adsorption capacity. Experiments investigating the effect of pH on adsorption capacity were conducted at a pH range of 2.0 to 10.0. The effect of initial phosphate concentration was determined at initial phosphate concentrations ranging between 0.5 mg L^−1^ to 100 mg L^−1^ and at a fixed pH of 4.0.

The adsorption capacity, q_t_, at a specific time *t* and the percent removal of phosphate were calculated based on the following equations:

1 $${q}_{t}=\frac{({C}_{0}-{C}_{t})V}{W}$$

2 $$ \% \,{\rm{removal}}=\frac{({C}_{0}-{C}_{t})}{{C}_{0}}\,\times \,{100} \% $$

here, *C*_0_ (mg L^−1^), and *C*_*t*_ (mg L^−1^) denote the initial and equilibrium phosphate concentrations, respectively, *V* (L) is the volume of the solution, and *W* (g) is the mass of the adsorbent used.

### Kinetics and Equilibrium Models

In order to evaluate the maximum phosphate uptake and identify the potential rate-controlling steps, three kinetic models (pseudo-first order, pseudo-second order, and intra-particle diffusion models) were applied to capture the adsorption process (on both HNT and 1Fe-HNT). The pseudo first order and pseudo second-order kinetics models are commonly used to obtain information on the equilibrium adsorption capacity of adsorbents. The model that provides the best fit and correlation coefficient is usually used to determine the adsorption capacity^34^. The details of these kinetic models are provided in the Supplementary Information.

The pseudo first order and pseudo second order kinetics models provide limited insights into the diffusion mechanism underlying this adsorption process. The Weber and Morris intraparticle diffusion model was used to identify the steps that occurred during the adsorption process and to elucidate whether intra-particle diffusion is the rate-limiting factor. The experimental data was therefore further tested against the Weber and Morris intra-particle diffusion model^35^ which can be expressed as follows:

3 $${{\rm{q}}}_{{\rm{t}}}={{\rm{k}}}_{{\rm{p}}}{{\rm{t}}}^{0.5}+{\rm{C}}$$

where, *q*_*t*_ (mg.g^−1^) is the amount of phosphate adsorbed at time *t* (min), *k*_*p*_ is the intraparticle diffusion rate constant (mg.g.min^0.5^) and *C* is a constant. The values of *k*_*p*_ and *C* can be determined from the intercept and slope of the linear plot of *q*_*t*_ versus *t*^*0.5*^.

In order to further understand the adsorption mechanism, classical adsorption isotherm models (i.e., Langmuir and Freundlich) were applied to fit the experimental data. These equilibrium models highlight sorbate-sorbent binding interaction and also give insights into possible mechanisms of adsorption. The Langmuir isotherm is based on monolayer adsorption onto the active sites^36^. An important characteristic of the Langmuir model is the dimensionless constant (*R*_*L*_), generally known as the separation factor, which was also estimated. The value of R_L_ indicates whether adsorption is irreversible (R_L_ = 0), favorable (0 < R_L_ < 1), linear (R_L_ = 1), or unfavorable (R_L_ > 1). The Freundlich isotherm, on the other hand, captures a non-ideal and reversible adsorption process not restricted to monolayer adsorption. This empirical model assumes a heterogeneous surface and that the amount adsorbed increases with sorbate concentration^37^. The model and its parameter details are outlined in the Supplementary Information. For maintaining consistency, all phosphate in the text is represented as orthophosphate (PO_4_).

## Results and Discussion

### Physical Morphology

**Figure 1:**
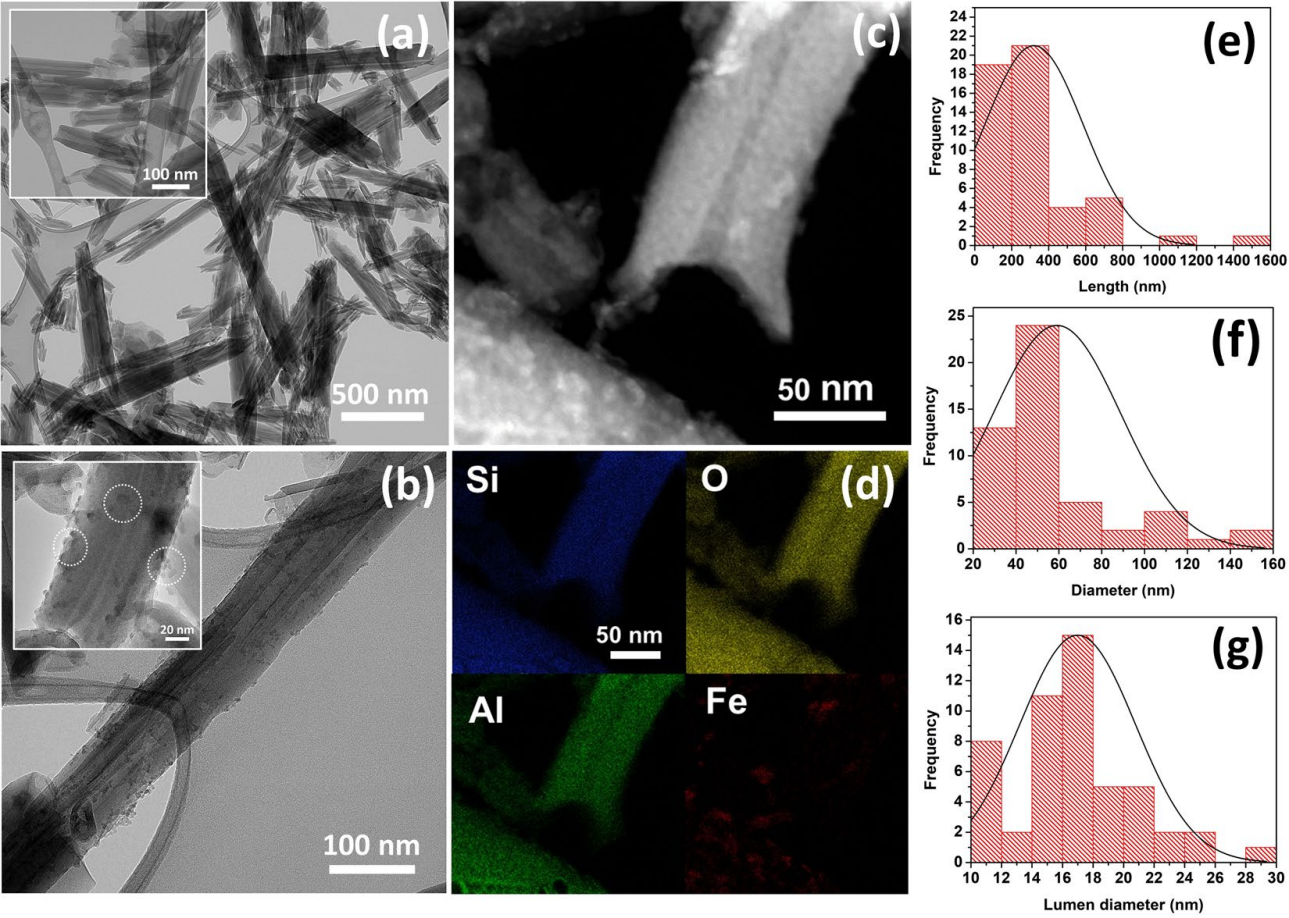
Representative TEM micrographs of (**a**) raw HNT and (**b**) 1Fe-HNT. (**c**,**d**) STEM element-specific images of 1Fe-HNT and (**e**) length, (**f**) diameter, (**g**) and lumen diameter distribution of HNTs.

SEM images of raw HNT and 1Fe-HNT (Fig. S2a,b) reveal the tubular structure of these clays. Clearer illustrations of the structure of the raw HNT and 1Fe-HNT are obtained through HRTEM imaging, as shown in Fig. 1a,b, respectively. 1Fe-HNT is chosen for TEM characterization since it is used for the equilibrium experiments. TEM images of the raw HNTs (Fig. 1a) illustrate the tubular structures of nanoclay, with open ends and a hollow cavity (lumen). A significant difference in HNT size, before and after modification (Fig. 1b), is not observed. For the 1Fe-HNTs shown in Fig. 1b, it is observed that the Fe_2_O_3_ nanoparticles are attached on the surface of the HNTs. The average diameter of the Fe_2_O_3_ nanoparticles on the surface of HNTs is determined to be 5.6 ± 0.92 nm.

STEM micrographs of 1Fe-HNT (Fig. 1c,d) confirm the presence of Fe, which is distributed throughout the surface of the HNTs. Since the surfaces of HNTs are negatively charged aluminosilicates, these could serve as polyanionic surfaces to allow for complex formation with iron cations^38^. The length of the raw HNTs ranges between 68 to 1520 nm (Fig. 1e), while the external diameter ranges between 20 to 150 nm (Fig. 1f). The lumen diameter is found to vary between 10 and 28 nm (Fig. 1g).

Relative to raw HNT, the specific surface area of the iron oxide modified HNTs is found to increase slightly from 64.4 to 70.5 m^2^/g (Table S1). This slight increase could be attributed to the contribution of nano-sized iron oxide particles, hybridized onto the HNTs. Figure S3 presents the N_2_ adsorption/desorption isotherms of HNT and modified HNTs at different iron oxide loadings. According to the classifications of International Union of Pure and Applied Chemistry (IUPAC), all isotherms of the raw and modified HNTs were of type II^39^ with H3 hysteresis loops^40^. The type II isotherm is indicative of a macroporous structure, however the hysteresis loop of the type H3 ascribes materials that have slit-shaped pores^41^.

### Chemical Composition and Crystallinity

Table S2 presents the chemical composition of the HNTs, evaluated with X-ray fluorescence. HNTs are primarily composed of silicon dioxide and aluminum dioxide with a trace amount of CaO, SrO, TiO_2_, phosphorous pentoxide, and sulfur trioxides. Raw HNTs contain a certain amount of Fe_2_O_3_ (0.59 wt.%), which increases with the increase in reagent loading. Iron content is also shown to increase upon hybridization. The amount of iron loading for 0.2, 0.5, 1, and 5Fe-HNT can be deduced from the table to be 0.92, 1.85, 2.47, and 5.98 wt.%, respectively.

**Figure 2:**
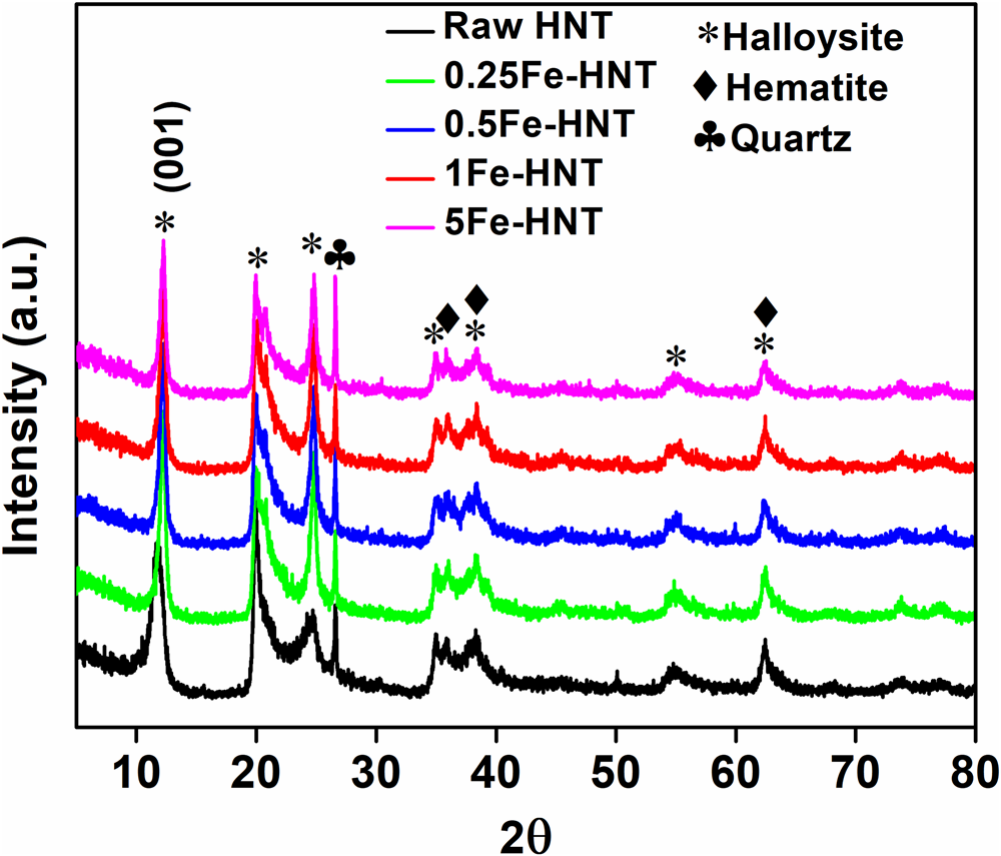
X-ray diffraction patterns of raw HNT and iron oxide modified HNT.

The XRD patterns of raw HNT and iron oxide modified HNTs are shown in Fig. 2. Clay minerals are primarily distinguished by the noticeable basal cleavage of the layered silicate structures. The first order basal reflection (001) for raw HNT is at 7.49Å, which is indicative of a kaolin-type mineral and of dehydrated halloysite^42^. The diffraction peaks at 11.8°, 19.9°, 24.7°, 35°, 38.3°, 54.9°, and 62.5° correspond to the d values of 7.49Å, 4.46Å, 3.60Å, 2.56Å, 2.35Å, 1.67Å, and 1.48Å, respectively, which can be indexed to raw HNT^43^. The diffraction peaks at 35.9°, 39.2, and 62.5° are attributed to hematite (α-Fe_2_O_3_)^44,45^. The presence of these peaks confirms the presence of iron oxides on the raw and modified HNTs.

### Surface Charge Density and Effect of pH on Adsorption Behavior

**Figure 3:**
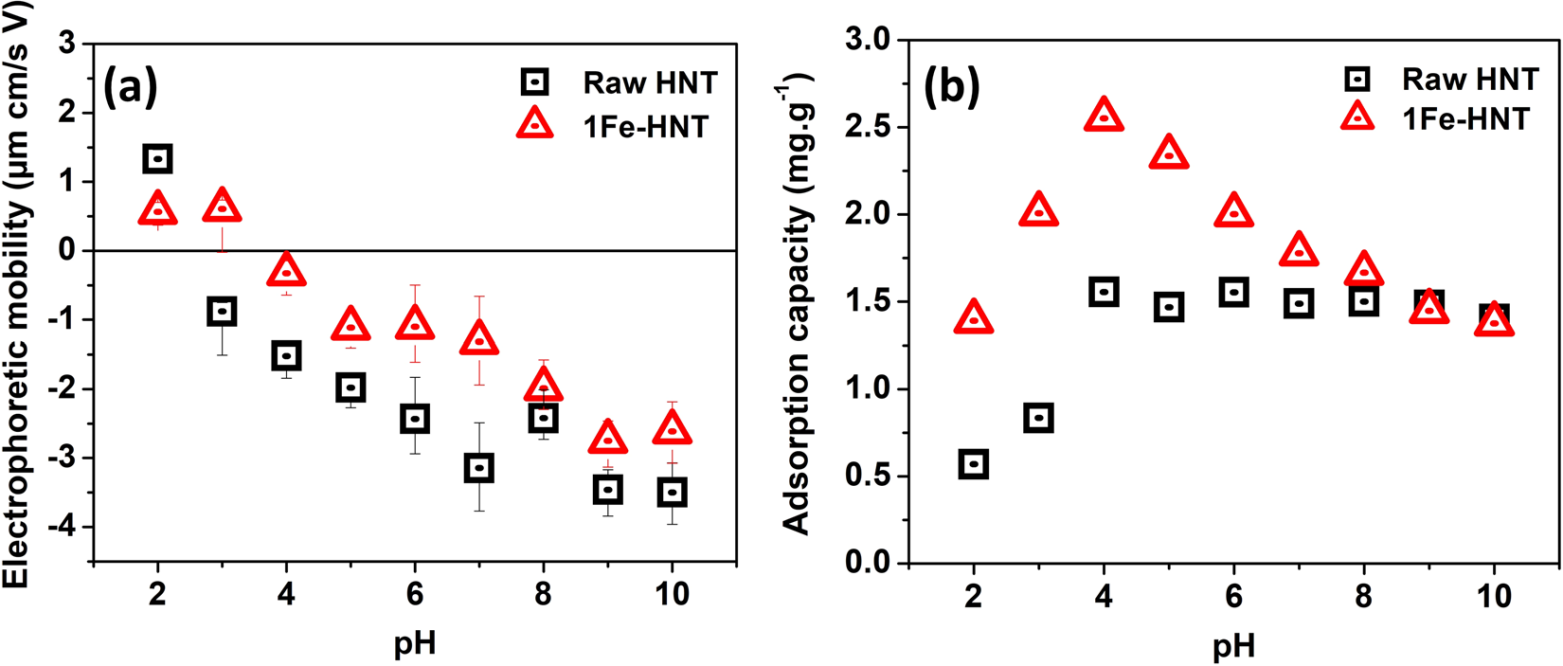
(**a**) Electrophoretic mobility and (**b**) adsorption capacity values as a function of pH for raw HNT and 1Fe-HNT. Initial phosphate concentration 10 mg L^−1^, contact time 240 min, sorbent dosage 3 g L^−1^, and shaking speed 240 rpm.

The electrophoretic mobility of HNT and 1Fe-HNT as a function of solution pH is shown in Fig. 3a. The positive charge at a low pH for raw HNT can arise from protonation of the hydroxyl groups on the clay edges or at surface defects sites^46^. The point of zero charge (PZC) for raw HNT is determined to be 2.5, which is consistent to the values reported earlier^47,48^. An interesting feature of HNT should be noted; i.e., the chemical composition difference between the exterior and interior of the HNTs, dominated by SiO_2_ (negatively charged) and Al_2_O_3_ (positively charged), respectively. It has been reported that the inner lumen consisting of Al_2_O_3_ maintains a positive charge throughout the pH range of 2.5 to 8.5^49^. This property allows for the selective adsorption of anions in the lumen in the pH range studied.

After modification of the HNTs with iron oxides, the PZC shifted to 3.3. Modifying HNT with iron oxide induces a shift towards a more positive value in mobility in most of the pH values studied, which is consistent to the previous observations with hematite-modified clay^50^. It is worth noting that the PZC of hematite is 5.8 as published earlier, suggesting that the hematite surfaces hold a positive charge over a wider pH range^50,51^. This phenomenon was also observed by Arias *et al*.^51^, and was attributed to the adsorption of hematite on the negatively charged silica basal surface of kaolinite resulting in the reduction of the number of negative charges on kaolinite^51,52^. The surface potential determination suggests that the reduction in the magnitude of the negative charge of 1Fe-HNT has likely occurred from the iron oxides bound to the HNT exterior; mutual charge neutralization probably have resulted between oppositely charged surface moieties (in HNTs and iron oxide)^50,51,53^. Another theory could be that the negative charges on the HNT are physically blocked by the iron oxides upon hybridization, indicating successful attachment of the iron oxides onto the HNT surfaces; a previous study involving hematite-kaolinite complexes reported a similar observation^54^.

Since iron dissolution at low pH can be a concern, dissolved iron content is analyzed to rule out such potential complication. Dissolution of SiO_2_ has predominantly been reported to take place at pH < 2.0 and at pH > 9.0^55^. Now, AlO_2_ is known to be relatively insoluble in acids and in strong alkali solutions^56,57^. Also, previous reports, investigating the effects of pH changes on hematite, conclude that the solubility of hematite is extremely low at pH > 3.0^58^. Experiments conducted at a pH of 2.0 with 1Fe-HNT and at a contact time of 240 min revealed the final iron concentration in the solution to be less than 0.05 mg/L, when filtered with a 0.45 µm filter; this indicates that the composite material maintains its integrity at a low pH.

The adsorption of phosphate onto raw HNT and 1Fe-HNT has been studied over a pH range of 2.0 to 10.0 (Fig. 3b). For raw HNT, the adsorption increased with an increase in pH up to 4.0, beyond which, the change in adsorption is insignificant. At pH 2.0, i.e., below the PZC of HNT, the dominant phosphate species are H_3_PO_4_ and H_2_PO_4_^−^, and are present in nearly equal concentrations. The adsorption of phosphate at these conditions can be attributed to electrostatic attraction between the positively charged sorbent exterior and negatively charged phosphate species. The low adsorption capacity could be attributed to a strong presence of uncharged H_3_PO_4_ species. However, at pH > PZC, the external surface of raw HNT is negatively charged (upon deprotonation) and the adsorption capacity is found to be 1.5 mg.g^−1^. This indicates that phosphate is likely been removed via electrostatic attraction between the anionic species and the positively charged inner lumen of HNTs.

**Figure 4:**
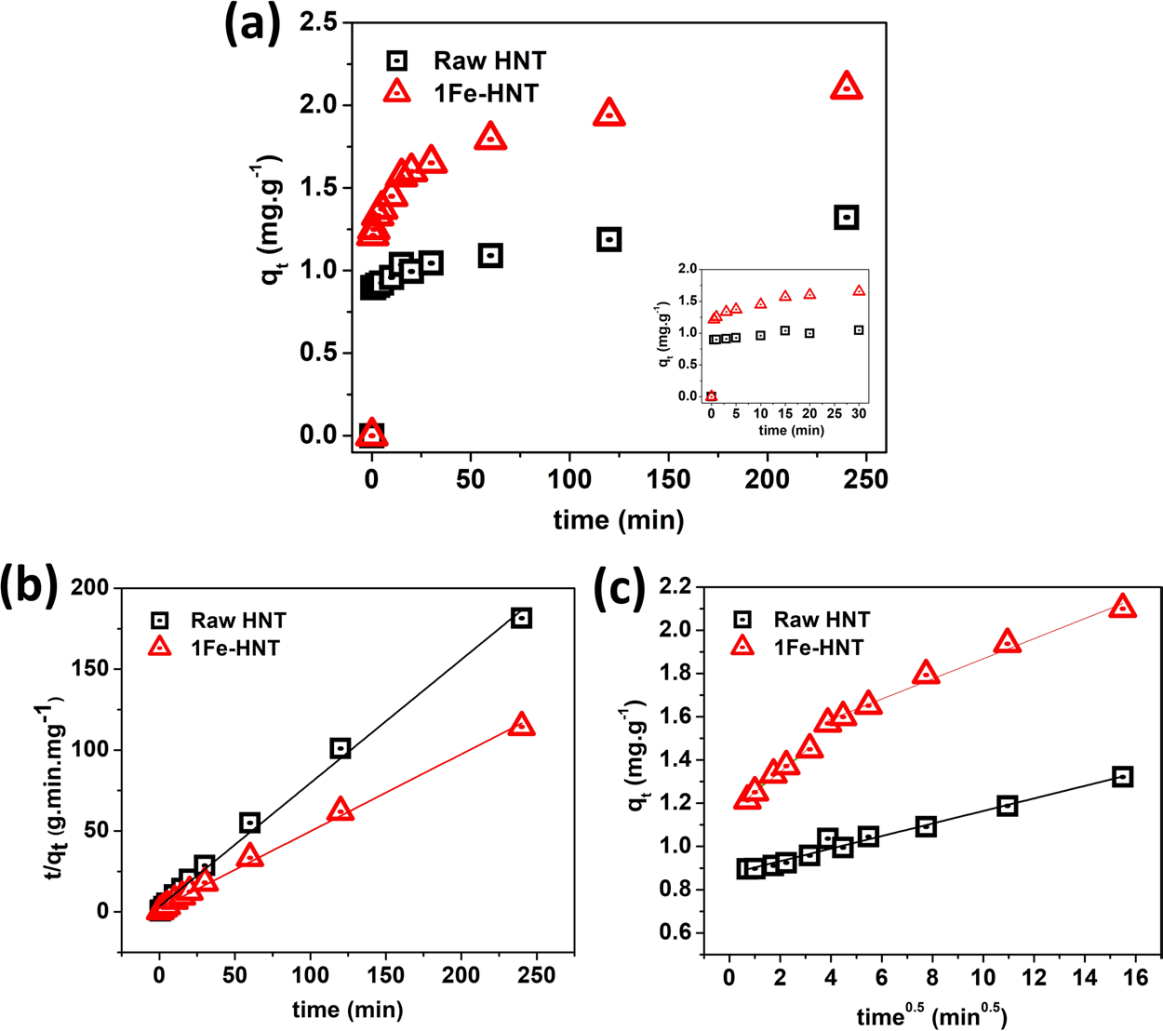
(**a**) Phosphate adsorption kinetics data plot for raw HNT and 1Fe-HNT and adsorption within first 30 min (inset). Modeling of phosphate adsorption kinetics with (**b**) pseudo-second order and (**c**) intraparticle diffusion models. Initial phosphate concentration: 10 mg L^−1^, pH 5.0, sorbent dosage 3 g L^−1^, shaking speed 240 rpm.

The change in pH affects the adsorption capacity of 1Fe-HNT sorbents in a unique way. It is known that hematite modification of kaolinite can induce a change in both its chemical and physical properties^27^. Adsorption of phosphate is observed to have occurred over the entire pH range (i.e., 2.0 through 10.0) for both HNT and 1Fe-HNT. HNTs possess a significant amount of hydroxyl groups on the edges^16^. Modification of HNTs with iron oxide nanoparticles increase the hydroxyl groups available on the surface and edges and thus can lead to an increase in the adsorption capacity of the sorbent. At pH 4.0, the dominant phosphate species is the mono-ionic H_2_PO_4_^−^ (98%). From Fig. 4 it appears that 1Fe-HNT and raw HNT both have an affinity for phosphate adsorption for this species. The underlying mechanism of such decrease in phosphate sorption at higher pH may be explained by the electrostatic repulsion between the negatively charged HNT or 1Fe-HNT exterior and the phosphate anions. Such repulsion has likely prevented interaction of the phosphate anions with the inner lumen of the clay, resulting in a decreased capacity. Furthermore, competition for adsorption sites between the OH^-^ ions and phosphate species may occur at an alkaline pH range^59^.

### The Kinetics of Adsorption

A significantly rapid adsorption is observed within the first 30 s (see inset of Fig. 4a) and equilibrium is reached in 4 h. The rapid adsorption in the initial stage can be attributed to the unique structure of the HNTs. As shown in the electron micrographs, HNTs have large pore diameters ranging between 10 to 30 nm, which can allow easy access of phosphate anions and binding in the inner lumen.

The kinetic data fit with pseudo-first order and pseudo-second order models are shown in Fig. 4b,c and the key parameters are listed in Table 1. It is known that the pseudo-first order model provides a better fit for the initial stage of the reaction process, specifically for those sorbents with rapid adsorption ability^60^. The correlation coefficient (*R*^2^) value for the pseudo-first order model is relatively lower than that for the pseudo-second order model. Furthermore, the calculated value of *q*_*e*,*calc*_ (mg.g^−1^) for 1Fe-HNT is significantly lower than the experimental value of *q*_*e*_,_*exp*_ (mg.g^−1^), obtained from the pseudo-first order model. This indicates that the pseudo-first order kinetic model is not suitable to capture the adsorption process.

**Table 1:** Kinetic parameters for phosphate adsorption onto HNT and 1Fe-HNT.

Kinetic models	Sorbents	
	Raw HNT	1Fe-HNT
*q*_*e*_,_*exp*_ (mg.g^−1^)	1.32	2.10
**Pseudo-first order**		
*q*_*e,calc*_ (mg.g^−1^)	1.13	0.14
*k*_*1*_ (min^−1^)	0.0691	0.0299
*R* ^2^	0.906	0.967
**Pseudo-second order**		
*q*_*e,calc*_ (mg.g^−1^)	1.31	2.10
*k*_2_ (g.mg^−1^.min^−1^)	0.174	0.107
*R* ^2^	0.995	0.997
**Intra-particle diffusion**		
*k*_*p*_ (mg.g.min^0.5^)	0.0290	
*C*	0.874	
*R* ^2^	0.980	
k_p1_ (mg.g.min^0.5^)		0.0959
*C*_1_		1.15
*R* ^2^		0.988
*k*_*p*2_ (mg.g.min^0.5^)		0.0465
*C*_2_		1.40
*R* ^2^		0.986

Conversely, the calculated equilibrium capacities for HNT and 1Fe-HNT obtained from the pseudo-second order model^61^ (Fig. 4b) are very similar to those estimated from the experimental analysis (as shown in Table 1). In addition, the higher correlation coefficients of raw HNT (*R*^2^ = 0.995) and 1Fe-HNT (*R*^2^ = 0.997) further suggest that the adsorption of phosphate onto HNTs and iron oxide modified HNTs follows the pseudo-second order model more closely. Similar results have been reported in the literature on adsorption of phosphate by illite^14^, kaolinite, montmorillonite, and hematite^62^.

A plot of q_t_ versus t^0.5^ yielding a linear relationship that passes through the origin indicates that intraparticle diffusion is the sole rate-limiting step in the reaction^35^. As shown in Fig. 4c, the linear plots for both HNT and 1Fe-HNT deviate from the origin; which indicate that intraparticle diffusion is involved in the adsorption process but is not the only rate-determining step. The value of *C* provides an insight onto the thickness of the boundary layer. In general, the larger the *C* value, the greater is the boundary layer effect (i.e., the greater is the contribution of surface adsorption in the rate-determining step), while a value of zero indicates that intra-particle diffusion dominates throughout the adsorption process. Also, higher values of *C* typically indicates higher adsorption^63^.

The q_t_ versus t^0.5^ plot of raw HNT shows the existence of a single rate while the plot for 1Fe-HNT shows two linear regimes; which implies a multi-stage adsorption process^14^. The initial linear regime is a result of external surface adsorption, driven by the initial phosphate concentration. The second linear regime signifies that phosphate species diffusing within the pores of the hollow HNT over time; which is typical in the case for intraparticle diffusion. This result is significant in that it provides further evidence for enhanced adsorption of phosphate on the exterior of the iron-oxide modified HNTs as opposed to that within the interior lumen (in case of raw HNTs). Table 1 shows that the rate constant for 1Fe-HNT in the first regime (*k*_*p1*_) is greater than the second (*k*_*p*2_); this indicates that intraparticle diffusion is the rate determining step of the entire adsorption process^64^.

### The Sorption Equilibrium Study

**Figure 5:**
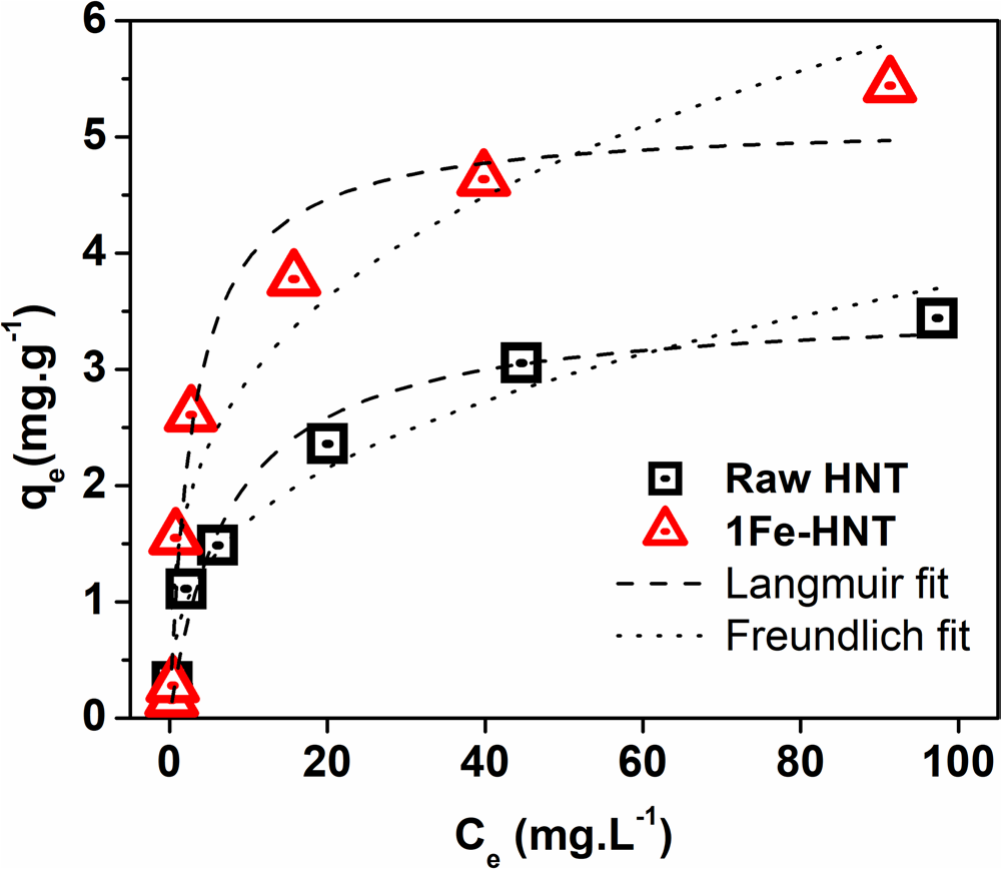
Effect of initial concentration on the adsorption capacity of raw HNT and 1Fe-HNT. Initial phosphate concentration: contact time 240 min, pH 4.0, sorbent dosage 3 g L^−1^, shaking speed 240 rpm.

The Langmuir and Freundlich isotherms are presented in Fig. 5 and the key parameters are listed in Table 2. The highest correlation coefficients for HNT (*R*^2^ = 0.973) and 1Fe-HNT (*R*^2^ = 0.955) are derived by fitting the equilibrium data with a Langmuir isotherm model. The monolayer capacities are near the experimental adsorption capacities, indicating an agreement with the Langmuir model. The calculated values for R_L_ of 0.070 and 0.029 for HNT and 1Fe-HNT, respectively; which confirm that the adsorption process is favorable^65^. These results indicate that the surfaces of HNTs and 1Fe-HNTs are relatively uniform and that a monolayer of phosphate coverage dominates the adsorption process. It has been observed in previous studies^66^ that anion adsorption at any pH value increases with the increase in sorbate concentration.

**Table 2:** Langmuir and Freundlich isotherm parameters for phosphate adsorption on HNT and 1Fe-HNT.

Equilibrium adsorption models	Sorbents	
	Raw HNT	1Fe-HNT
q_e,exp_ (mg.g^−1^)	3.43	5.46
**Langmuir**		
*X*_*m*_ (mg.g^−1^)	3.56	5.13
*b*	0.133	0.339
*R*_*L*_	0.070	0.029
*R* ^2^	0.973	0.955
**Freundlich**		
K_F_ (mg g^−1^(dm^3^/g)^n^)	0.769	1.42
*1/n*	0.342	0.312
*R* ^*2*^	0.952	0.904

The Freundlich isotherm model showed lower correlation coefficient values but *R*^*2*^ values were greater than 0.90 for both sorbents (Table 2). The Freundlich constant K_F_ is associated with the adsorption capacity of the sorbent; a higher value indicates a higher affinity for the sorbate^64^.

### Effect of Coexisting Anions

**Figure 6:**
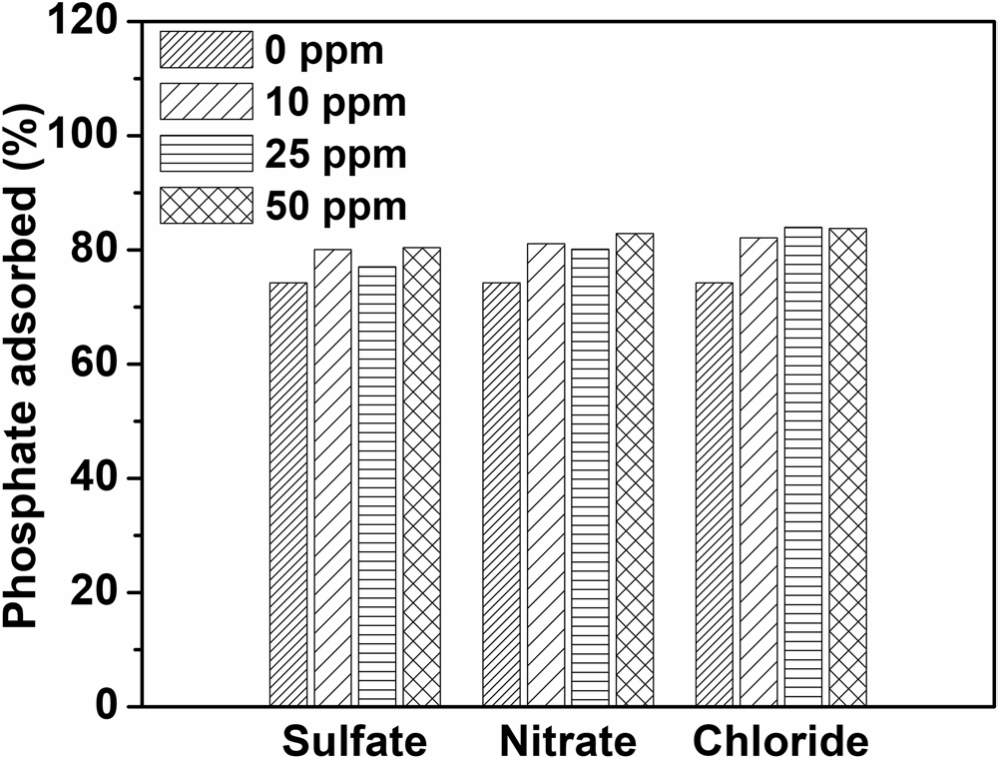
Effect of common coexisting ions on the adsorption capacity of Fe-HNT. Initial phosphate concentration: 10 mg L^−1^, contact time 240 min, pH 4.0, sorbent dosage 3 g L^−1^, shaking speed 240 rpm.

In water and wastewater, anions such as sulfate, nitrate, and chloride coexist with phosphate ions and will likely compete for adsorption sites. The effect of these anions on the adsorption capacity is investigated and the results are shown in Fig. 6. It is observed that the addition of the coexisting anions in the solution does not impede the removal of phosphate from water, rather slightly enhance it.

Although the presence of anions in a solution has generally been reported to compete for adsorption sites, the total adsorption capacity of some metal oxides has been found to be unaffected or has increased in some cases^25,67^. Specific additives, such as NaCl, NaNO_3_, and KNO_3_ have been found to enhance phosphate removal^25^. It can also be inferred that chloride may contribute in the adsorption of phosphate from the solution^25^. A similar phenomenon was reported by Zhang and coworkers^68^ and Giesler *et al*.^29^ who used other metal oxides for phosphate adsorption. It is thus deduced that anions that adsorb via outer-sphere complexation, are highly sensitive to ionic strength. Consequently, the adsorption of these anions is inhibited by competition with other weakly adsorbing coexisting anions. On the contrary, anions that adsorb by inner-sphere complexation either display little sensitivity to ionic strength (or competing ligands) or respond to increasing ionic strength with increased adsorption^29,68,69^. Chubar and coworkers^70^ attributed this phenomenon to intermediate complex formation by chloride anions (by replacing the surface hydroxyl anions) with metal oxides; such complexation reduce the energy required for chelation between H_2_PO_4_^−^ and metal oxide surfaces^25,70^.

### Adsorption Mechanisms

The effect of pH on the surface charge and adsorption capacity of the sorbent reveals vital information on the sorption mechanism. To answer the question of why the sorbent with a negative surface charge has an affinity for anion sorption, it is important to address the concepts of both specific and non-specific adsorption. The results from the pH studies indicate an occurrence of specific adsorption (ligand exchange or chemisorption); i.e., formation of a strong covalent bond due to the attachment of the ion directly to the surface without a water molecule interposed in between^71^. Furthermore, the chemical component of the adsorption free energy dominates in specific adsorption; as a result, sorption occurs on positive, negative, or neutral surfaces^66,71^. On the other hand, when non-specific adsorption (i.e. electrostatic attraction) governs the adsorption process, the sorbent surface must have a positive overall charge in order for phosphate sorption to occur; consequently, adsorption can only occur at a pH lower than the PZC^66^, which is not the case in this study. Furthermore, at the low pH conditions, an increase in the final pH value is observed (Table S3). This indicates that phosphate adsorption is likely caused by exchange of ions; i.e., an exchange between the phosphate and the hydroxide ligands on the surface occurred, which released hydroxides into the solution. Also, ligand exchange can occur in conjunction with electrostatic attraction between the negatively charged phosphate anion and positively charged surfaces (for pH < PZC) and the inner lumen^27,66^.

**Figure 7:**
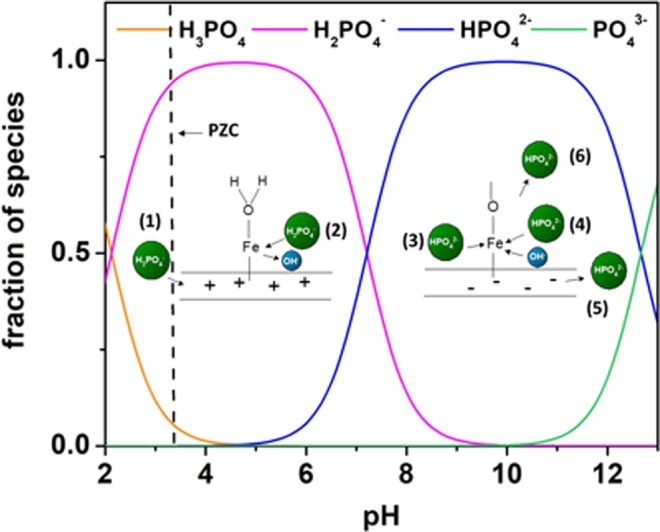
Phosphate sorption mechanism on HNTs modified with iron oxide nanoparticles. The mechanisms that can occur include (1) electrostatic attraction, (2) ligand exchange, (3) Lewis acid-base interactions, (4) competing hydroxyl anions, and (5) and (6) electrostatic repulsion.

However, at an alkaline pH, the observed decrease in equilibrium pH can result from the deprotonation of the coordinated water molecules on the active site. Adsorption can occur via Lewis acid-base interaction in which the iron active site becomes a weak base (Lewis base) and the phosphate anions act as a weak acid (Lewis acid)^8,72^. Furthermore, during this interaction, a reactive iron site can react with oxygen in the phosphate anions to develop Fe-O coordination bonds, thus releasing an H_2_O molecule in the process as shown below. This mechanism was also proposed in earlier studies with iron(hydr)oxide sorbents^73^. Figure 7 provides a graphic illustrating the mechanisms of phosphate adsorption onto Fe-HNTs.

The complexation mechanism to describe the reaction of phosphate ions on raw HNT and iron oxide modified HNT is adopted from an earlier study^27^, conducted for surface complexation of phosphate ions on hematite-kaolinite surfaces.

4 $${\rm{SOH}}+{{\rm{H}}}^{+}\leftrightarrow {{{\rm{SOH}}}_{{\rm{2}}}}^{+}$$

5 $${\rm{SOH}}\leftrightarrow {{\rm{SO}}}^{-}+{{\rm{H}}}^{+}$$

6 $${\rm{SOH}}+{{\rm{H}}}_{{\rm{3}}}{{\rm{PO}}}_{{\rm{4}}}\leftrightarrow {{\rm{SH}}}_{{\rm{2}}}{{\rm{PO}}}_{{\rm{4}}}+{{\rm{H}}}_{{\rm{2}}}{\rm{O}}$$

7 $${\rm{SOH}}+{{\rm{H}}}_{{\rm{3}}}{{\rm{PO}}}_{{\rm{4}}}\leftrightarrow {{{\rm{SHPO}}}_{{\rm{4}}}}^{-}+{{\rm{H}}}_{{\rm{2}}}{\rm{O}}+{{\rm{H}}}^{+}$$

8 $${\rm{SOH}}+{{\rm{H}}}_{{\rm{3}}}{{\rm{PO}}}_{{\rm{4}}}\leftrightarrow {{{\rm{SPO}}}_{{\rm{4}}}}^{{\rm{2}}-}+{{\rm{H}}}_{{\rm{2}}}{\rm{O}}+{{\rm{2H}}}^{+}$$

where, SOH denotes the surface functional group of halloysite and iron oxide modified halloysite.

### Sorption Performance Comparison with Other Sorbents

Table 3 lists a comparison of equilibrium and maximum adsorption capacities of Fe-HNT and other adsorbents that are considered low-cost, naturally available, or that exhibit similar chemical features. In general, the adsorption capacity of Fe-HNT is similar to or exceeds that of other low-cost adsorbents, such as iron hydr(oxide) coated sand, magnetic iron oxide nanoparticles, and other clays^74–76^. Although the achieved adsorption capacity in this study is lower than that of modified activated carbon, HNTs have a much lower cost compared to granular activated carbon and commercial activated carbon (Table 4). Sand, on the other hand, is low in cost, however, its adsorption capacity towards phosphate is poor. The time to reach adsorption equilibrium is an important factor, which can dictate the feasibility of an adsorbent for a treatment process; Fe-HNT shows a relatively short equilibrium time, relative to the other adsorbents.

**Table 3:** Phosphate adsorption capacities of other clay and iron (modified) sorbents.

Adsorbent	Equilibrium adsorption capacity (mg/g)	Maximum adsorption capacity (mg/g)	Equilibrium time (h)	pH	Reference
Iron oxide coated GAC	~7.0	~10.8	100	6.5	^78^
Iron hydr(oxide) coated sand	0.385	0.693	24	7	^74^
Ferrihydrite	3.8	8.248	100	5	
Kaolinite	~0.25	0.28	24	7–8	^75^
Montmorillonite	1.22	2.29	24	7.6	^75^
Halloysite	1.32	3.56	4	4	This study
Geothite	___	6.63	24	7	^79^
Magnetic iron oxide nanoparticles	4.93	5.02	24	__	^76^
Fe-HNT	2.10	5.46	4	4	This study

**Table 4:** Cost of some of the compared sorbents.

Adsorbent	Cost (USD/kg)	Reference
Granular activated carbon	1.44	^80^
Commercial activated carbon	4.00	^81^
Iron oxide	2.07	^82^
Sand	0.009	^83^
Kaolinite	0.12	^84^
Montmorillonite	0.044–0.36	^84^
Halloysite	0.2–0.5	^81, 85^
1Fe-HNT	0.33–0.63	This study

Considering the low cost and comparable adsorption capacity of Fe-HNT, this sorbent can be considered as a promising material for phosphate removal from water. Furthermore, its natural and environmentally friendly features make it suitable to be used as a fertilizer or soil conditioner. Selling the phosphate saturated adsorbent material as a fertilizer could be more cost effective than regenerating it; since the cost of regeneration usually account for more than 70% of the total operating and maintenance cost of an adsorption system^77^. However, future work should involve the study of regenerating the material with NaOH and precipitating and retrieving the phosphate from the solution using Ca(OH)_2_.

## Conclusions

In this study, a facile preparation of a natural and environmentally benign iron oxide modified halloysite nanotubular clay (1Fe-HNT) is presented. TEM micrographs, diffractograms, and XRF spectrographs confirm successful hybridization of nano-scale iron oxide (α-Fe_2_O_3_) onto HNT surfaces. Relative to raw HNT, the as-prepared nanosorbent displays an enhanced adsorption capacity (for phosphate). The adsorption capacity is found to be pH-dependent. Kinetics results reveal that the prepared sorbent can remove more than 30% of the phosphate within the first 30 s. The kinetics behavior closely follows a pseudo-second order process, and intraparticle diffusion is likely the rate-limiting step. A Langmuir model best describes the adsorption isotherm data. The presence of coexisting ions slightly increase the adsorption capacity of phosphate for 1Fe-HNT, specifically when chloride is present. This indicates strong selectivity of the sorbents towards phosphates. It can be deduced that electrostatic attraction and ligand exchange govern sorption at low pH, while Lewis acid-base interactions dominate the process as the pH increases. The results presented in this study demonstrate that 1Fe-HNT is a promising sorbent for phosphate removal from water. Furthermore, this study provides promising results for the use of HNTs and Fe-HNTs in ceramic filters as their size at the nano-scale range provides added advantage over other larger-scale sorbents.

## Supplementary information

Adsorption of phosphate on iron oxide doped halloysite nanotubes

## Data Availability

Additional data that support the findings of this study are available in supplementary information and from the corresponding author upon request.
