# Supplementary material for: Adsorption of phosphate on iron oxide doped halloysite nanotubes
Source: Sci Rep. 2019 Mar 1;9:3232. doi: 10.1038/s41598-019-39035-2 (PMC6397243; doi:10.1038/s41598-019-39035-2)
Supplement: Supplementary file 1 — Adsorption of phosphate on iron oxide doped halloysite nanotubes [file 41598_2019_39035_MOESM1_ESM.docx]

**Electronic Supplementary Information**

**Adsorption of phosphate on iron oxide doped halloysite nanotubes**

*Dema A. Almasri,^a,b^ Navid B. Saleh,^c^ Muataz A. Atieh,^a,b*^* Gordon McKay,^b^ *and Said Ahzi ^a,b*^*

^a^ Qatar Environment and Energy Research Institute (QEERI), Hamad Bin Khalifa University (HBKU), Qatar Foundation, PO Box 34110, Doha, Qatar

^b^ College of Science and Engineering, Hamad Bin Khalifa University, Qatar Foundation, PO Box, 34110, Doha, Qatar

^c^ Department of Civil, Architectural and Environmental Engineering, University of Texas, Austin, TX 78712, U.S.A.

*Corresponding authors:

Muataz A. Atieh

E-mail: mhussein@hbku.edu.qa

Said Ahzi

E-mail: [sahzi@hbku.edu.qa](mailto:sahzi@hbku.edu.qa)

**Effect of iron oxide loading and adsorbent dosage**

The effect of the adsorbent dose on the removal of phosphate can be found in Figure S1a which also indicates the percent removal of phosphate at different iron oxide loadings. It can be observed that 3 g L^-1^ of the modified HNT was a sufficient amount for quantitative adsorption of phosphate. The amount of phosphate increased with increasing sorbent dosage. An enhancement in adsorption was not significant at the high dosage amount of 8 g L^-1^ indicating that nearly all phosphate ions in the solution were adsorbed. Since adsorption on the iron modified HNTs is more enhanced with increasing sorbent dosage relative to raw HNTs, it is evident that the iron oxides played a significant role in providing adsorption sites for phosphate species. However, there did not appear to be a noteworthy enhancement in adsorption capacity between 1Fe-HNT and 5Fe-HNT. Figure S1b depicts the amount (in mg) of phosphate adsorbed onto the sorbent per gram of sorbent. The amount of phosphate adsorbed per gram of sorbent decreases with increasing sorbent dose.

*
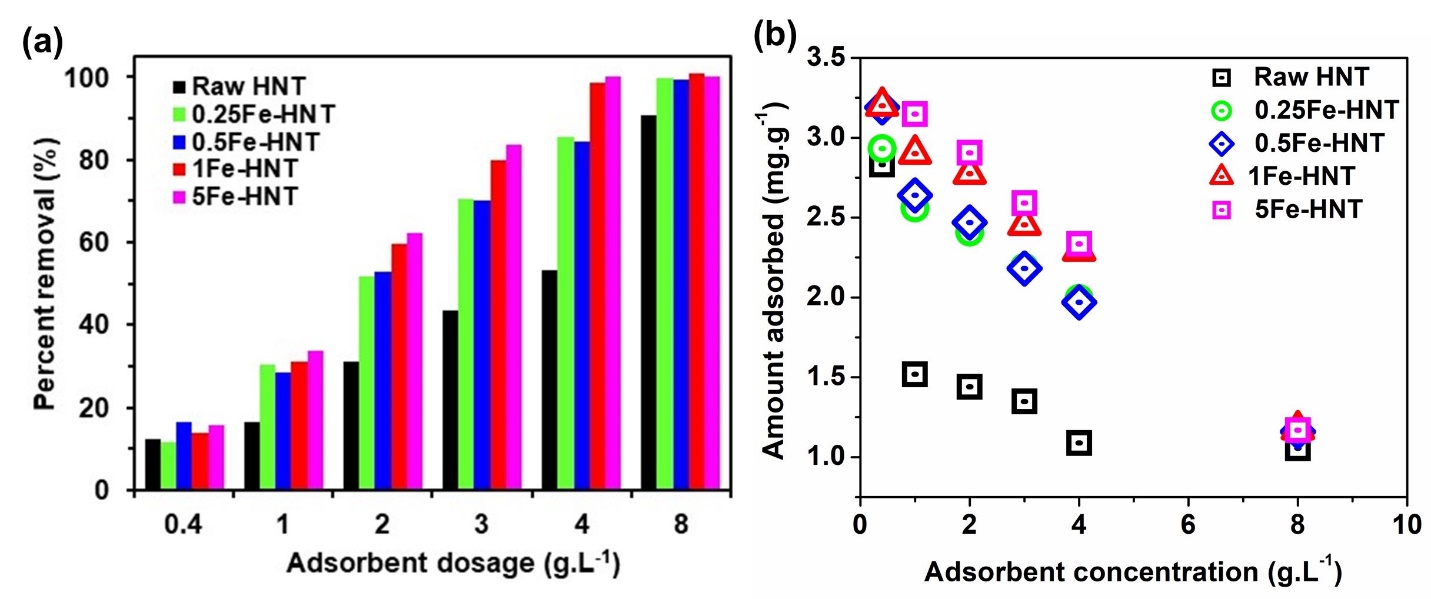
*

**Figure S1.** Effect of adsorbent dosage on phosphate removal depicted in percent removal (a) and amount adsorbed per gram of sorbent. Initial phosphate concentration: 10 mg L^-1^, pH 5, contact time 120 min, shaking speed 350 rpm.

Therefore, from an economical and practical standpoint, the 1Fe-HNT at 3 g L-1 was selected as the optimum sorbent and dosage amount, respectively. Since these experiments were conducted as screening tests, the kinetics of the reaction was not yet obtained and, thus, the dosage experiments were conducted at a contact time of 120 min. It should be mentioned that the adsorbent dosage experiments were conducted in a laboratory different than the rest of the experiments and a slight difference in phosphate removal was observed. This was attributed to the different deionized water used and ion chromatography instruments. The dosage experiments served as a guide for us to select the most efficient adsorbent dosage and iron loading to be used in the proceeding batch experiments in comparison with raw HNT.

**Kinetic models**

The pseudo-first order ^1^ equation can be expressed as follows:

$\log\left( q_{e}-q_{t} \right)=\log\left( q_{e} \right)-\frac{k_{1}t}{2.303}$ (1)

where, q­_e_ and q_t_ (mg.g^-1^) depict the phosphate adsorption capacity at equilibrium and at time t (min), respectively, and k­_1_ (min^-1^) depicts the pseudo-first order rate constant. The parameters q_e_ and k_1_ can be determined from the slope and intercept of the plot of log(q_e_-q_t_) versus t.

The pseudo-second order ^2^ equation can be expressed as follows:

$\frac{t}{q_{t}}=\frac{1}{k_{2}q_{e}^{2}}+\frac{t}{q_{e}}$ (2)

where, *q_t_* (mg.g^-1^) is the amount of phosphate adsorbed at a certain time t (min), *q_e_* (mg g^-1^) is the amount of phosphate adsorbed at equilibrium, and *k_2_* (g. mg^-1^.min^-1^) is the pseudo-second-order reaction rate. From the intercept and slope of the ( *t/q_t_*) vs. *t* plot, *k_2_* and *q_e_* can be determined, respectively.

**Adsorption isotherm models**

The nonlinear form of the Langmuir isotherm is expressed in the equation as follows:

$q_{e}=\frac{X_{m}bC_{e}}{1+bC_{e}}$ (3)

where, *q_e_* and C_e_ are the amount of phosphate adsorbed per unit mass of adsorbent material (mg g^-1^) and the equilibrium concentration of phosphate (mg.L^-1^), respectively. *X_m_* and *b* are Langmuir constants representing the monolayer capacity (mg.g^-1^) and equilibrium constant, respectively.

The dimensionless constant (*R_L_*), generally known as the separation factor, was calculated also. as shown below ^3^:

$R_{L}=\frac{1}{1+bC_{0}}$ (4)

where, C_0_ is the highest initial concentration (mg.L^-1^).

The non-linear form of the Freundlich model is expressed as:

$q_{e}=K_{f}C_{e}^{1/n}$ (5)

where, *q_e_* and *C_e_* are the amount of phosphate adsorbed per unit mass of adsorbent material (mg.g^-1^) and the equilibrium concentration of phosphate (mg.L^-1^), respectively. *K_f_* and *n* are Freundlich constants related to the adsorption capacity and intensity, respectively.

**Scanning electron microscopy**

As shown in Figure S2, a morphological difference in the HNTs was not observed between the raw HNT and iron modified HNT. In order to obtain a more clear structural analysis of the nanotubular clay and iron oxide modification, high resolution TEM imaging was done.


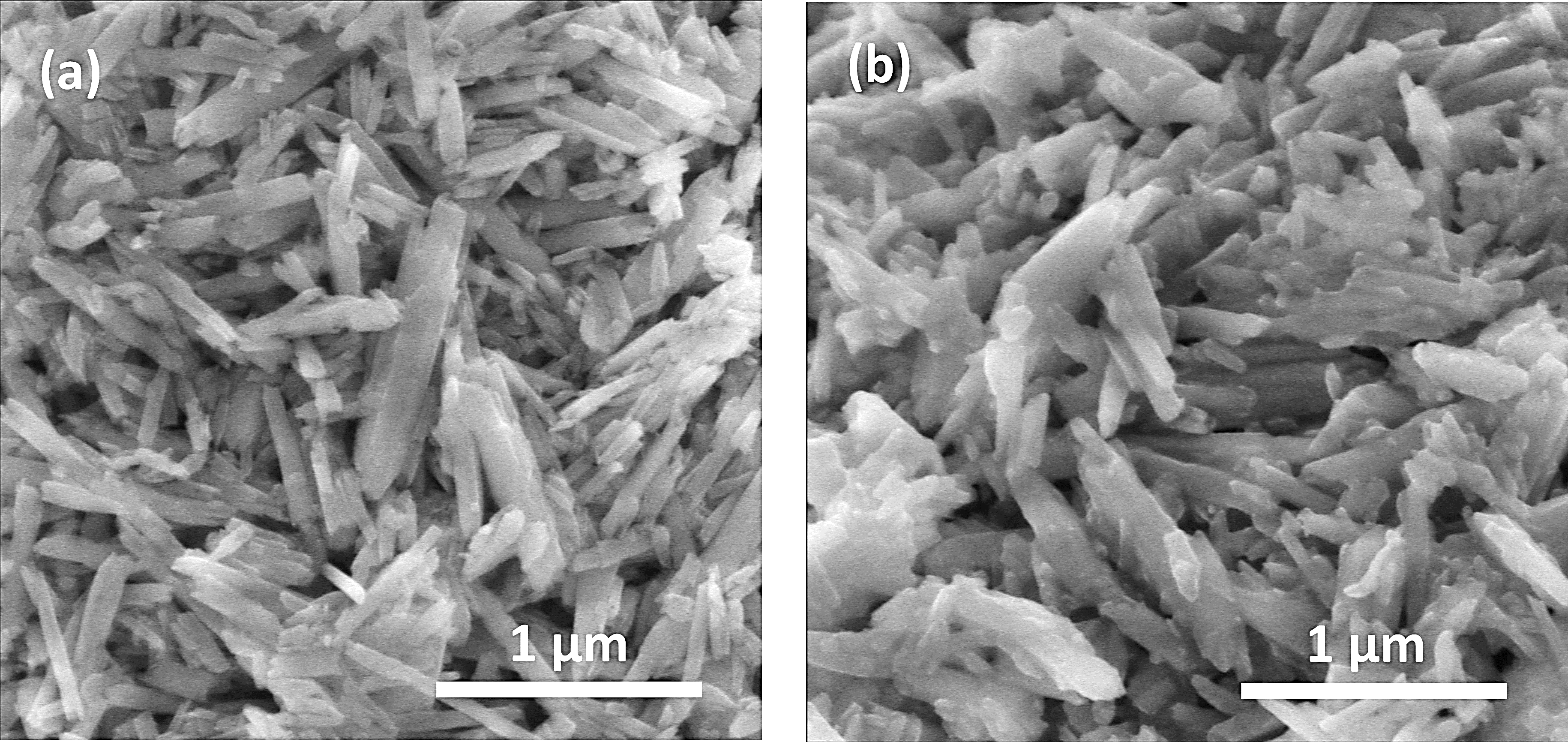


**Figure S2.** SEM images of raw HNT (a) and 1Fe-HNT (b).

**Surface area analysis**

Figure S3 illustrates the N_2_ adsorption/desorption isotherms of HNT and modified HNTs at different iron oxide loadings. A “knee” is present between 0 to 0.1 P/P^0^ relative pressure which indicates monolayer coverage is complete and multilayer adsorption is about to initiate^4^.

**Table S1.** BET surface area of raw HNT and modified HNT.

| Sample | BET specific surface area (m^2^ g^-1^) |
| --- | --- |
| Raw HNT | 64.43 |
| 0.25Fe-HNT | 70.24 |
| 0.5Fe-HNT | 75.60 |
| 1Fe-HNT | 70.46 |
| 5Fe-HNT | 70.16 |





**Figure S3.** N_2_ adsorption-desorption isotherms for raw HNT and iron oxide modified HNTs (solid line-adsorption, dashed line- desorption).

**Chemical composition (X-ray Fluorescence analysis)**

**Table S2.** Chemical composition of the raw and iron oxide modified halloysite samples obtained by XRF analysis.

|  | Compound (wt.%) | | | | | | | | | |
| --- | --- | --- | --- | --- | --- | --- | --- | --- | --- | --- |
| Sample | **SiO_2_** | **Al_2_O_3_** | **Fe_2_O_3_** | **P_2_O_5_** | **SO_3_** | **CaO** | **SrO** | | **TiO_2_** | **Sum** |
| HNT-raw | 53.8 | 43.8 | 0.59 | 0.69 | 0.30 | 0.18 | | 0.11 | 0.06 | 99.5 |
| 0.25Fe-HNT | 53.3 | 43.5 | 1.51 | 0.69 | 0.32 | 0.12 | | 0.11 | 0.05 | 99.6 |
| 0.5Fe-HNT | 52.9 | 43 | 2.44 | 0.70 | 0.27 | 0.12 | | 0.11 | 0.06 | 99.6 |
| 1Fe-HNT | 52.4 | 42.9 | 3.06 | 0.63 | 0.27 | 0.1 | | 0.11 | 0.05 | 99.5 |
| 5Fe-HNT | 50.3 | 41.5 | 6.57 | 0.72 | 0.21 | 0.12 | | 0.11 | 0.06 | 99.6 |

**Initial and final pH after adsorption**

**Table S3.** Initial pH and final pH after adsorption.

| \| **Raw HNT** \| \|  \| **1Fe-HNT** \| \| \| --- \| --- \| --- \| --- \| --- \| \| **Initial pH** \| **Final pH** \|  \| **Initial pH** \| **Final pH** \| \| 2.13 \| 2.32 \|  \| 2.14 \| 2.18 \| \| 2.89 \| 2.81 \|  \| 3.07 \| 3.24 \| \| 3.98 \| 4.27 \|  \| 4.08 \| 4.52 \| \| 5.02 \| 4.99 \|  \| 4.99 \| 5.03 \| \| 6.05 \| 5.37 \|  \| 6.02 \| 5.35 \| \| 6.92 \| 5.53 \|  \| 6.98 \| 5.92 \| \| 8.09 \| 6.09 \|  \| 8.03 \| 6.78 \| \| 9.03 \| 6.67 \|  \| 9.13 \| 6.42 \| \| 9.98 \| 6.87 \|  \| 9.92 \| 6.82 \| |
| --- | --- | --- | --- | --- | --- | --- | --- | --- | --- | --- | --- | --- | --- | --- | --- | --- | --- | --- | --- | --- | --- | --- | --- | --- | --- | --- | --- | --- | --- | --- | --- | --- | --- | --- | --- | --- | --- | --- | --- | --- | --- | --- | --- | --- | --- | --- | --- | --- | --- | --- | --- | --- | --- | --- | --- |

**Literature Cited**

1 Yuh-Shan, H. Citation review of Lagergren kinetic rate equation on adsorption reactions. *Scientometrics* **59**, 171-177 (2004).

2 Ho, Y.-S. & McKay, G. Pseudo-second order model for sorption processes. *Process Biochemistry* **34**, 451-465 (1999).

3 Jiang, J.-Q., Cooper, C. & Ouki, S. Comparison of modified montmorillonite adsorbents: Part I: preparation, characterization and phenol adsorption. *Chemosphere* **47**, 711-716, doi:<http://dx.doi.org/10.1016/S0045-6535(02)00011-5> (2002).

4 Sing, K. S. & Williams, R. T. Physisorption hysteresis loops and the characterization of nanoporous materials. *Adsorption Science & Technology* **22**, 773-782 (2004).
